# Supplementary material for: Correlations of the Gastric and Duodenal Microbiota with Histological, Endoscopic, and Symptomatic Gastritis
Source: J Clin Med. 2019 Mar 5;8(3):312. doi: 10.3390/jcm8030312 (PMC6462939; doi:10.3390/jcm8030312)
Supplement: Supplementary file 1 [file jcm-08-00312-s001.pdf]

## SUPPLEMENTARY

### Supplementary Table 1. Species with a relative abundance of >0.10% found in the antrum, body, and duodenum

| Phylum | Antrum (*n* = 98) | | Body (*n* = 98) | | Duodenum (*n* = 98) | |
|---|---|---|---|---|---|---|
| | Species | % | Species | % | Species | % |
| Proteobacteria | *Helicobacter pylori* | 21.43 | *Helicobacter pylori* | 26.08 | *Brevundimonas aurantiaca* | 42.89 |
| | *Dechloromonas sp.* | 5.01 | *Pseudomonas veronii* | 5.00 | *Methylobacterium longum* | 2.56 |
| | *Pseudomonas veronii* | 2.97 | *Dechloromonas sp.* | 2.43 | *Dechloromonas sp.* | 0.93 |
| | *Pseudomonas sp.* | 1.12 | *Pseudomonas sp.* | 1.71 | *Sphingomonas echinoides* | 0.92 |
| | *Haemophilus parainfluenzae* | 0.85 | *Haemophilus sp.* | 1.50 | *Cupriavidus necator* | 0.74 |
| | *Hydrogenophilus hirschii* | 0.82 | *Escherichia coli* | 1.24 | *Helicobacter pylori* | 0.72 |
| | *Moraxella osloensis* | 0.58 | *Moraxella osloensis* | 1.11 | *Pseudomonas grimontii* | 0.63 |
| | *Escherichia coli* | 0.64 | *Moraxella sp.* | 0.78 | *Bradyrhizobium pachyrhizi* | 0.59 |
| | *Acinetobacter johnsonii* | 0.53 | *Haemophilus parainfluenzae* | 0.54 | *Schlegelella thermodepolymerans* | 0.55 |
| | *Haemophilus sp.* | 0.49 | *Enhydrobacter aerosaccus* | 0.51 | *Stenotrophomonas maltophilia* | 0.49 |
| | *Paracoccus sp.* | 0.43 | *Hydrogenophilus hirschii* | 0.48 | *Tepidimonas fonticaldi* | 0.47 |
| | *Brevundimonas sp.* | 0.38 | *Paracoccus sp.* | 0.45 | *Escherichia coli* | 0.46 |
| | *Sphingobacterium multivorum* | 0.38 | *Variovorax paradoxus* | 0.37 | *Massilia sp.* | 0.32 |
| | *Pantoea sp.* | 0.37 | *Brevundimonas sp.* | 0.36 | *Hydrogenophilus hirschii* | 0.31 |
| | *Sphingomonas sp.* | 0.36 | *Sphingomonas sp.* | 0.31 | *Haemophilus parainfluenzae* | 0.31 |
| | *Sphingomonas dokdonensis* | 0.36 | *Azospirillum sp.* | 0.21 | *Paracoccus sp.* | 0.29 |
| | *Azospirillum sp.* | 0.34 | *Sphingobacterium multivorum* | 0.21 | *Neisseria perflava* | 0.28 |
| | *Neisseria mucosa* | 0.33 | *Paracoccus aminovorans* | 0.19 | *Moraxella osloensis* | 0.20 |
| | *Moraxella sp.* | 0.33 | *Neisseria mucosa* | 0.18 | *Bradyrhizobium denitrificans* | 0.20 |
| | *Methylobacterium isbiliense* | 0.33 | *Actinobacillus parahaemolyticus* | 0.16 | *Neisseria flavescens* | 0.12 |
| | *Massilia sp.* | 0.33 | *Acinetobacter sp.* | 0.13 | *Actinobacillus parahaemolyticus* | 0.11 |
| | *Enhydrobacter aerosaccus* | 0.30 | *Caulobacter henricii* | 0.12 | | |
| | *Methylobacterium sp.* | 0.24 | *Tepidiphilus succinatimandens* | 0.12 | | |
| | *Herbaspirillum sp.* | 0.22 | *Neisseria elongata* | 0.11 | | |
| | *Nevskia ramosa* | 0.21 | | | | |
| | *Paracoccus aminovorans* | 0.21 | | | | |
| | *Methylobacterium adhaesivum* | 0.18 | | | | |
| | *Methylobacterium jeotgali* | 0.17 | | | | |
| | *Acinetobacter schindleri* | 0.15 | | | | |
| | *Neisseria elongata* | 0.13 | | | | |

| | | | | | | |
|---|---|---|---|---|---|---|
| | *Campylobacter showae* | 0.12 | | | | |
| Actinobacteria | *Propionibacterium acnes* | 7.27 | *Propionibacterium acnes* | 9.42 | *Propionibacterium acnes* | 15.02 |
| | *Corynebacterium vitaeruminis* | 1.86 | *Corynebacterium vitaeruminis* | 1.08 | *Corynebacterium tuberculostearicum* | 1.67 |
| | *Corynebacterium sp.* | 0.89 | *Propionibacterium granulosum* | 0.74 | *Corynebacterium vitaeruminis* | 1.01 |
| | *Dermacoccus sp.* | 0.85 | *Dermacoccus sp.* | 0.77 | *Corynebacterium accolens* | 0.58 |
| | *Propionibacterium granulosum* | 0.74 | *Corynebacterium segmentosum* | 0.51 | *Corynebacterium sp.* | 0.32 |
| | *Corynebacterium macginleyi* | 0.45 | *Corynebacterium macginleyi* | 0.49 | *Propionibacterium granulosum* | 0.21 |
| | *Corynebacterium segmentosum* | 0.42 | *Corynebacterium sp.* | 0.40 | *Actinomyces odontolyticus* | 0.15 |
| | *Corynebacterium mastitidis* | 0.31 | *Corynebacterium doosanense* | 0.26 | *Rothia mucilaginosa* | 0.15 |
| | *Geodermatophilus sp.* | 0.26 | *Corynebacterium aurimucosum* | 0.20 | *Dermacoccus sp.* | 0.12 |
| | *Corynebacterium mucifaciens* | 0.24 | *Actinomyces graevenitzii* | 0.17 | | |
| | *Actinomyces sp.* | 0.15 | *Corynebacterium mastitidis* | 0.13 | | |
| | *Propionibacterium thoenii* | 0.14 | *Rhodococcus erythropolis* | 0.11 | | |
| | *Arthrobacter sp.* | 0.13 | | | | |
| | *Dermabacter hominis* | 0.13 | | | | |
| Bacteroidetes | *Cloacibacterium rupense* | 14.27 | *Cloacibacterium rupense* | 10.73 | *Cloacibacterium rupense* | 6.15 |
| | *Prevotella melaninogenica* | 1.75 | *Prevotella melaninogenica* | 2.25 | *Prevotella melaninogenica* | 1.29 |
| | *Sediminibacterium sp.* | 0.98 | *Sediminibacterium sp.* | 1.60 | *Sediminibacterium sp.* | 0.77 |
| | *Prevotella pallens* | 0.77 | *Prevotella nanceiensis* | 0.67 | *Porphyromonas endodontalis* | 0.38 |
| | *Prevotella nanceiensis* | 0.43 | *Prevotella pallens* | 0.64 | *Prevotella pallens* | 0.26 |
| | *Porphyromonas endodontalis* | 0.43 | *Porphyromonas endodontalis* | 0.53 | *Porphyromonas catoniae* | 0.25 |
| | *Prevotella intermedia* | 0.41 | *Porphyromonas gingivalis* | 0.36 | *Capnocytophaga granulosa* | 0.17 |
| | *Prevotella copri* | 0.38 | *Prevotella sp.* | 0.35 | *Prevotella histicola* | 0.16 |
| | *Capnocytophaga gingivalis* | 0.35 | *Prevotella copri* | 0.30 | *Alloprevotella rava* | 0.14 |
| | *Porphyromonas gingivalis* | 0.24 | *Capnocytophaga gingivalis* | 0.24 | *Prevotella copri* | 0.14 |
| | *Alloprevotella rava* | 0.24 | *Alloprevotella rava* | 0.22 | *Capnocytophaga sputigena* | 0.13 |
| | *Porphyromonas catoniae* | 0.23 | *Capnocytophaga sputigena* | 0.20 | *Bacteroides sp.* | 0.13 |
| | *Chryseobacterium hominis* | 0.20 | *Cloacibacterium normanense* | 0.19 | *Prevotella nanceiensis* | 0.13 |
| | *Bacteroides sp.* | 0.19 | *Capnocytophaga granulosa* | 0.17 | *Prevotella nigrescens* | 0.12 |
| | *Prevotella sp.* | 0.19 | *Tannerella forsythia* | 0.16 | | |
| | *Chryseobacterium aquaticum* | 0.14 | *Prevotella nigrescens* | 0.13 | | |
| | *Flavobacterium succinicans* | 0.13 | *Chryseobacterium hominis* | 0.12 | | |
| | *Prevotella tannerae* | 0.13 | | | | |
| | *Prevotella nigrescens* | 0.13 | | | | |
| | *Cloacibacterium normanense* | 0.12 | | | | |
| | *Flavobacterium sp.* | 0.12 | | | | |
| Firmicutes | *Staphylococcus epidermidis* | 2.17 | *Staphylococcus epidermidis* | 2.89 | *Bacillus sp.* | 4.30 |
| | *Streptococcus mitis* | 1.94 | *Bacillus sp.* | 2.60 | *Staphylococcus epidermidis* | 1.67 |

| | | | | | | |
|---|---|---|---|---|---|---|
| | *Bacillus sp.*<br>*Peptoniphilus sp.*<br>*Streptococcus infantis*<br>*Streptococcus sp.*<br>*Streptococcus anginosus*<br>*Streptococcus salivarius* | 1.73<br>0.84<br>0.56<br>0.30<br>0.17<br>0.13 | *Streptococcus mitis*<br>*Peptoniphilus sp.*<br>*Streptococcus sp.*<br>*Streptococcus infantis*<br>*Enterococcus cecorum*<br>*Mycoplasma faucium*<br>*Finegoldia sp.* | 1.67<br>1.42<br>0.62<br>0.24<br>0.16<br>0.14<br>0.12 | *Staphylococcus hominis*<br>*Peptoniphilus sp.*<br>*Enterococcus cecorum*<br>*Streptococcus sp.* | 0.46<br>0.43<br>0.36<br>0.15 |
| Fusobacteria | *Fusobacterium periodonticum*<br>*Fusobacterium nucleatum*<br>*Leptotrichia genomosp.*<br>*Leptotrichia sp.* | 0.98<br>0.69<br>0.36<br>0.19 | *Fusobacterium periodonticum*<br>*Fusobacterium nucleatum*<br>*Leptotrichia genomosp.*<br>*Leptotrichia sp.* | 0.98<br>0.55<br>0.50<br>0.21 | *Fusobacterium periodonticum*<br>*Fusobacterium nucleatum*<br>*Leptotrichia genomosp.* | 0.56<br>0.33<br>0.19 |
| Other phyla | *Deinococcus caeni* | 0.18 | *Treponema sp.* | 0.12 | None | - |

**Supplementary Table 2. Relative abundance of significant species and updated Sydney system scores according to the presence of regular arrangement of collecting venules**

| Common species and pathology findings | | Regular arrangement of collecting venules (*n* = 62) | Irregular arrangement of collecting venules (*n* = 36) | *p*-value |
|---|---|---|---|---|
| Antrum | *Helicobacter pylori* | 0.03 (0 - 50.0) | 74.85 (0 - 94.0) | < 0.001 |
| | *Pseudomonas veronii* | 3.75 (0 - 14.2) | 0.29 (0 - 9.9) | < 0.001 |
| | *Pseudomonas sp.* | 1.15 (0 - 5.6) | 0.07 (0 - 3.8) | < 0.001 |
| | *Dechlomonas sp.* | 4.20 (0 - 38.4) | 0.99 (0 - 17.4) | 0.009 |
| | *Propionibacterium acnes* | 9.70 (0 - 27.3) | 1.95 (0 - 13.8) | < 0.001 |
| | *Cloacibacterium rupense* | 14.65 (0 - 76.5) | 6.15 (0.3 - 42.8) | 0.009 |
| | *Staphylococcus epidermidis* | 1.80 (0 - 31.6) | 0.06 (0 - 6.2) | 0.004 |
| | *Streptococcus mitis* | 0.15 (0 - 63.2) | 0.04 (0 - 5.5) | 0.108 |
| | *Bacillus sp.* | 0.35 (0 - 32.6) | 0.10 (0 - 15.8) | 0.310 |
| | *Variovorax paradoxus* | 0.02 (0 - 1.0) | 0.01 (0 - 0.01) | 0.180 |
| | *Porphyromonas gingivalis* | 0.07 (0 - 4.0) | 0.11 (0 - 3.0) | 0.016 |
| | Neutrophil (0 : 1 : 2 : 3) | 4 : 55 : 3 : 0 | 0 : 9 : 21 : 6 | < 0.001 |
| | Mononuclear cell (0 : 1 : 2 : 3) | 60 : 1 : 0 : 1 | 10 : 11 : 12 : 3 | < 0.001 |
| | Atrophy (0 : 1 : 2 : 3) | 38 : 24 : 0 : 0 | 17 : 19 : 0 : 0 | 0.176 |
| | Intestinal metaplasia (0 : 1 : 2 : 3) | 62 : 0 : 0 : 0 | 31 : 4 : 1 : 0 | 0.011 |
| Body | *Helicobacter pylori* | 0.43 (0 - 27.0) | 89.40 (0 - 98.0) | < 0.001 |
| | *Pseudomonas veronii* | 5.93 (0 - 25.1) | 0.29 (0 - 14.3) | < 0.001 |
| | *Pseudomonas sp.* | 2.23 (0 - 12.2) | 0.08 (0 - 4.2) | < 0.001 |
| | *Dechlomonas sp.* | 1.70 (0 - 22.5) | 0.06 (0 - 3.9) | < 0.001 |
| | *Propionibacterium acnes* | 12.30 (0 - 29.1) | 1.48 (0 - 23.1) | < 0.001 |
| | *Cloacibacterium rupense* | 7.55 (0 - 66.4) | 1.45 (0 - 54.9) | 0.002 |
| | *Staphylococcus epidermidis* | 3.15 (0 - 15.9) | 0.10 (0 - 26.8) | 0.010 |
| | *Streptococcus mitis* | 0.19 (0 - 34.9) | 0.03 (0 - 4.4) | 0.022 |
| | *Bacillus sp.* | 1.50 (0 - 32.0) | 0.05 (0 - 6.0) | 0.002 |
| | *Variovorax paradoxus* | 0.09 (0 - 29.0) | 0.14 (0 - 4.9) | 0.552 |
| | *Porphyromonas gingivalis* | 0.11 (0 - 4.2) | 0.16 (0 - 6.1) | 0.024 |
| | Neutrophil (0 : 1 : 2 : 3) | 1 : 60 : 0 : 1 | 0 : 12 : 21 : 3 | < 0.001 |
| | Mononuclear cell (0 : 1 : 2 : 3) | 61 : 0 : 0 : 1 | 10 : 6 : 19 : 1 | < 0.001 |
| | Atrophy (0 : 1 : 2 : 3) | 37 : 25 : 0 : 0 | 25 : 11 : 0 : 0 | 0.334 |
| | Intestinal metaplasia (0 : 1 : 2 : 3) | 62 : 0 : 0 : 0 | 35 : 1 : 0 : 0 | 0.367 |

Using the Kruskal-Wallis test, the relative abundance of species is presented as median values with range (minimum - maximum). Using the Fisher's exact test, pathologcal findings are shown as the numbers of subjects with no (score 0), mild (score 1), moderate (score 2), and marked (score 3) degrees based on the updated Sydney classification system.

**Supplementary Table 3. Differences in PAGI-SYM scores according to endoscopic findings**

| Endoscopic findings / Symptom subscales | Hemorrhagic spots | | Thickened rugae | | Advanced atrophy | | Mucosal nodularity | | Regular arrangement of collecting venues | |
|---|---|---|---|---|---|---|---|---|---|---|
| | Present (*n* = 6) | Absent (*n* = 92) | Present (*n* = 7) | Absent (*n* = 91) | Present (*n* = 9) | Absent (*n* = 89) | Present (*n* = 13) | Absent (*n* = 85) | Present (*n* = 62) | Absent (*n* = 36) |
| Heartburn & regurgitation | **17.0 (8 - 19)** | **7.5 (0 - 23)** | 10.0 (0 - 22) | 7.8 (0 - 23) | 5.7 (0 - 20) | 8.2 (0 - 23) | 9.0 (0 - 20) | 7.8 (0 - 23) | 7.6 (0 - 23) | 9.0 (0 - 22) |
| Bloating | **6.0 (5 - 14)** | **3.6 (0 - 10)** | 4.3 (0 - 10) | 3.7 (0 - 14) | 4.4 (0 - 7) | 3.7 (0 - 14) | 5.0 (1 - 14) | 3.6 (0 - 10) | 3.3 (0 - 9) | 4.6 (0 - 14) |
| Nausea & vomiting | 6.5 (4 - 10) | 3.2 (0 - 11) | 3.7 (0 - 9) | 3.4 (0 - 11) | 5.0 (0 - 11) | 3.4 (0 - 10) | 5.4 (0 - 11) | 3.1 (0 - 10) | 3.0 (0 - 10) | 4.6 (0 - 11) |
| Upper abdominal pain | 5.7 (4 - 8) | 4.2 (0 - 9) | 4.0 (0 - 7) | 4.4 (0 - 9) | 4.0 (0 - 8) | 4.5 (0 - 9) | 5.0 (2 - 8) | 4.4 (0 - 9) | 4.2 (0 - 9) | 4.6 (0 - 8) |
| Fullness & early satiety | 7.0 (2 - 11) | 4.2 (0 - 15) | 5.0 (0 - 9) | 4.3 (0 - 15) | 5.0 (0 - 9) | 4.3 (0 - 15) | 6.5 (0 - 12) | 3.9 (0 - 15) | 3.6 (0 - 15) | 5.5 (0 - 12) |
| Lower abdominal pain | **5.7 (1 - 8)** | **1.8 (0 - 8)** | 4.3 (0 - 6) | 1.9 (0 - 8) | 1.4 (0 - 8) | 2.1 (0 - 8) | 3.0 (0 - 8) | 1.9 (0 - 8) | 1.6 (0 - 8) | 3.1 (0 - 8) |

PAGI-SYM scores are shown as median values with ranges (minimum - maximum) using the Kruskal–Wallis test. For each subscales, statistical significance was set at $p$ <0.0083 ($p$ <0.05 divided by 6 subscales) after multiple testing correction. Significant scores are rendered in boldface. PAGI-SYM, Patient Assessment of Gastrointestinal Disorders Symptom Severity Index.

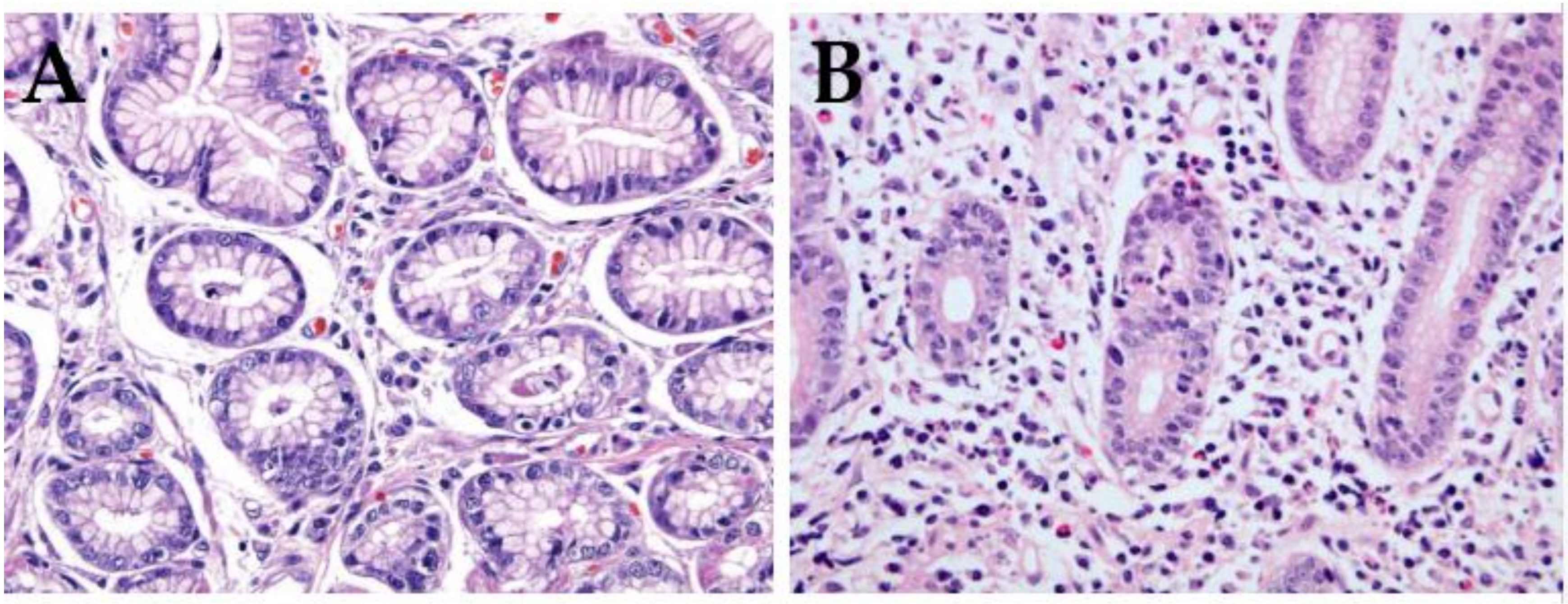


Figure S1: Microscopic findings of the biopsy specimens
